# Reassessing chest compression sites in pediatric cardiopulmonary resuscitation without ventilatory support using echocardiographic assessment: a prospective observational study

**DOI:** 10.1038/s41598-025-22823-4

**Published:** 2025-11-06

**Authors:** Dongbum Suh, Jin Hee Lee, Hyuksool Kwon, Mi Jin Kim

**Affiliations:** 1 Department of Emergency Medicine, Seoul National University Bundang Hospital, Seoul National University College of Medicine, 82, Gumi-ro 173beon-gil, Bundang-gu, Seongnam-si, Gyeonggi-do 13620 Republic of Korea; 2 Research Center for Disaster Medicine, Seoul National University College of Medicine, Seoul, Republic of Korea; 3 Department of Emergency Medicine, Seoul National University Hospital, Seoul, Republic of Korea

**Keywords:** Cardiopulmonary resuscitation, Chest compression, Pediatrics, Respiratory cycle, Point-of-care ultrasound, Paediatric research, Echocardiography

## Abstract

The recommended chest compression site during pediatric cardiopulmonary resuscitation (CPR) is based on studies that did not account for dynamic respiratory changes in cardiac position. Since cardiac position changes with respiratory phase, the optimal site during CPR without respiratory support, which physiologically corresponds to the end-expiration state, may differ. We assessed heart position at end-expiration using echocardiography. A prospective observational study was conducted in 18 children under 7 years of age. Transthoracic echocardiography was performed to identify the position of the left ventricle during both inspiration and expiration. Each subject underwent five measurements in each respiratory phase. The position of the left ventricle was recorded relative to the inter-nipple line and categorized by intercostal space (ICS). At end-expiration, the left ventricle was commonly located at the first upper ICS (53.3%) and nipple line (27.8%), with only 4.4% located at the first lower ICS and none below that level. During inspiration, the left ventricle was located at the first lower ICS (50.0%) and nipple line (35.6%). Linear mixed-effects regression demonstrated a significant upward shift in heart position during expiration compared to inspiration (β = -1.46; 95% CI, -1.91 to -1.00; *p* < 0.001), independent of age, sex, and weight. These findings confirm that respiratory phase affects heart position in young children, with expiration associated with consistent cephalad shift. Therefore, the optimal chest compression site during pediatric CPR without respiratory support may be more cephalad than current guideline recommendations, suggesting the need to re-evaluate traditional landmarks in light of physiologic cardiac motion.

**Supplementary Information:**

The online version contains supplementary material available at 10.1038/s41598-025-22823-4.

## Introduction

Survival to hospital discharge following in-hospital pediatric cardiac arrest has improved over the past two decades, largely due to early recognition, high-quality cardiopulmonary resuscitation (CPR), post-cardiac arrest care, and the implementation of extracorporeal cardiopulmonary resuscitation^1–3^. In contrast, survival to discharge following out-of-hospital pediatric cardiac arrest remained largely unchanged, with rates ranging from approximately 6.7% to 10.2% between 2007 and 2012^4–6^.

High-quality CPR remains the cornerstone of resuscitative efforts, with key components including delivery of chest compressions at an appropriate rate and depth, minimization of interruptions in chest compressions, complete chest recoil between compressions, and avoidance of excessive ventilation^7^. The proper hand placement during compressions is essential to ensure optimal cardiac output and improve survival outcomes. Optimal cardiac output during CPR is achieved by direct compression of the left ventricular chamber, allowing maximal stroke volume generation. Compression over the left ventricular outflow tract (LVOT) may paradoxically reduce cardiac output by obstructing flow, emphasizing the critical importance of accurate hand placement targeting the main ventricular chamber^8^.

According to the 2020 American Heart Association guidelines, the recommended chest compression site in infants and children is just below the intermammary line^7^. This recommendation is based on studies from 1980 s to 2000, which utilized radiological assessments, physiologic hemodynamic studies during chest compressions, and anthropometric analyses to determine the optimal compression site in pediatric patients^9–12^.

Recent studies in adults have raised questions about the accuracy of current compression landmarks. Studies using various imaging modalities and clinical outcome data have demonstrated that the actual position of cardiac structures often differs from guideline-recommended compression site^13,14^, Furthermore, investigations examining the relationship between compression location and hemodynamic outcomes have suggested that alternative compression sites may produce superior blood pressure^15^. These adult findings highlight the need for similar reassessment in pediatric populations, where anatomical variations and the dynamic nature of cardiac position may have even greater clinical significance.

While recent adult studies have increasingly explored lateral displacement from the sternal midline to optimize compression over the left ventricle^15–17^, pediatric studies have primarily focused on the vertical axis^18,19^. These studies have assessed whether landmarks such as the inter-nipple line or the lower third of the sternum adequately align with the left ventricle, and some have also investigated potential injury risks associated with these compression sites. However, one significant limitation of the previous studies is that they did not account for the impact of the respiratory phase on cardiac position. During cardiac arrest, the respiratory state is presumed to approximate end-expiration, as depletion of adenosine triphosphate leads to relaxation of the respiratory muscles, including the diaphragm, and progressive collapse of the lungs below functional residual capacity, thereby creating a physiologic condition consistent with the end-expiratory phase^20^. Recent analyses have relied on static imaging modalities such as chest X-ray and computed tomography, which inherently do not reflect the dynamic movement of thoracic structures during the respiratory cycle. As a result, these studies may not reflect the true anatomical relationships during CPR^15–19^.

The position of the heart changes during inspiration and expiration, and studies in adults have demonstrated that the heart descends by approximately 4.9 to 28.0 mm during inspiration compared to expiration^21,22^. However, the extent and clinical significance of these positional changes in pediatric patients remain unclear.

**Fig. 1:**
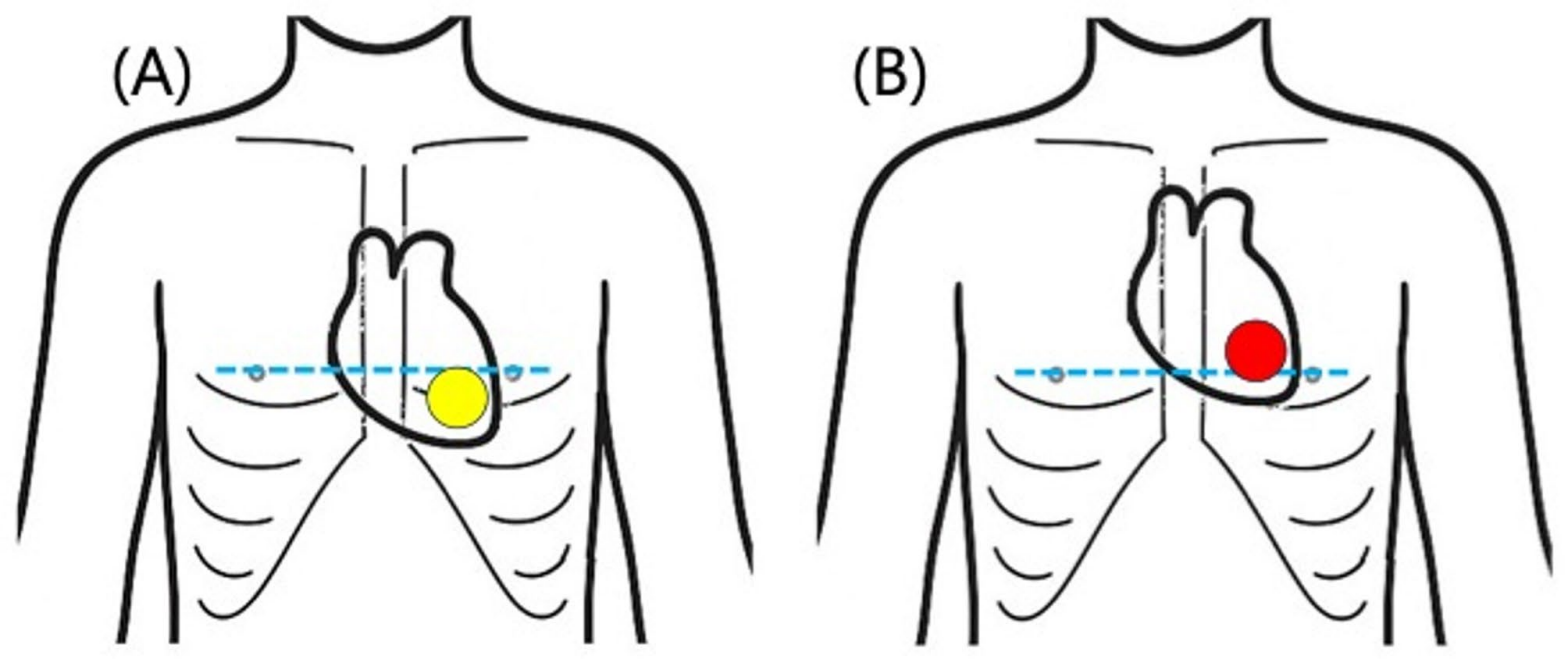
Conceptual illustration of the hypothesized changes in left ventricular position in the pediatric thorax during inspiration and expiration. **(A)** Inspiration; **(B)** Expiration. The position of the left ventricle shifts during the respiratory cycle, descending below the inter-nipple line during inspiration (yellow circle) and ascending to the level of or above the inter-nipple line during expiration (red circle). The blue line indicates the inter-nipple line.

This study aimed to identify and characterize the dynamic changes in left ventricular position throughout the respiratory cycle using transthoracic echocardiography in pediatric patients. While the findings are most directly applicable to compression-only CPR scenarios, which mainly comprise bystander-initiated pediatric out-of-hospital cardiac arrest, we also present data on inspiratory positions to inform understanding of cardiac position during ventilated CPR (Fig. 1).

## Materials and methods

### Study design and data source

This prospective observational study was conducted from May 2018 to June 2023 in the pediatric emergency department (PED) of Seoul National University Bundang Hospital, a tertiary academic medical center and designated regional emergency center. The study was temporarily suspended during the COVID-19 pandemic, from February 2020 to April 2022, due to institutional restrictions and limited access to non-emergent echocardiographic examinations in pediatric patients.

The study protocol was approved by the Institutional Review Board of Seoul National University Bundang Hospital (B-1804-465-302), and all procedures were performed in accordance with relevant laws, institutional guidelines, and the ethical standards outlined in the Declaration of Helsinki. Informed consent was obtained from the legal guardians of all participants prior to enrollment.

### Selection of participants

The study enrolled pediatric patients under 7 years of age who presented to the PED during the study period.

Patients with congenital heart defects (e.g., coarctation of the aorta, pulmonary stenosis, tetralogy of Fallot, transposition of the great arteries), significant chest wall or thoracic structural deformities (e.g., pectus excavatum, pectus carinatum, spinal deformity), mediastinal abnormalities (e.g., mediastinal mass), a history of chest trauma or thoracic surgery, or hemodynamic instability, as well as those with conditions that could impair normal thoracic motion, including recent respiratory diseases (e.g., pneumonia, asthma, atelectasis, pneumothorax, or hemothorax), diaphragmatic abnormalities, or severe chest pain, were excluded from the study.

### Measurements

For each participant, demographic and clinical information including sex, age, body weight, and chief complaint was recorded at the time of enrollment.

### Echocardiographic assessment

**Fig. 2:**
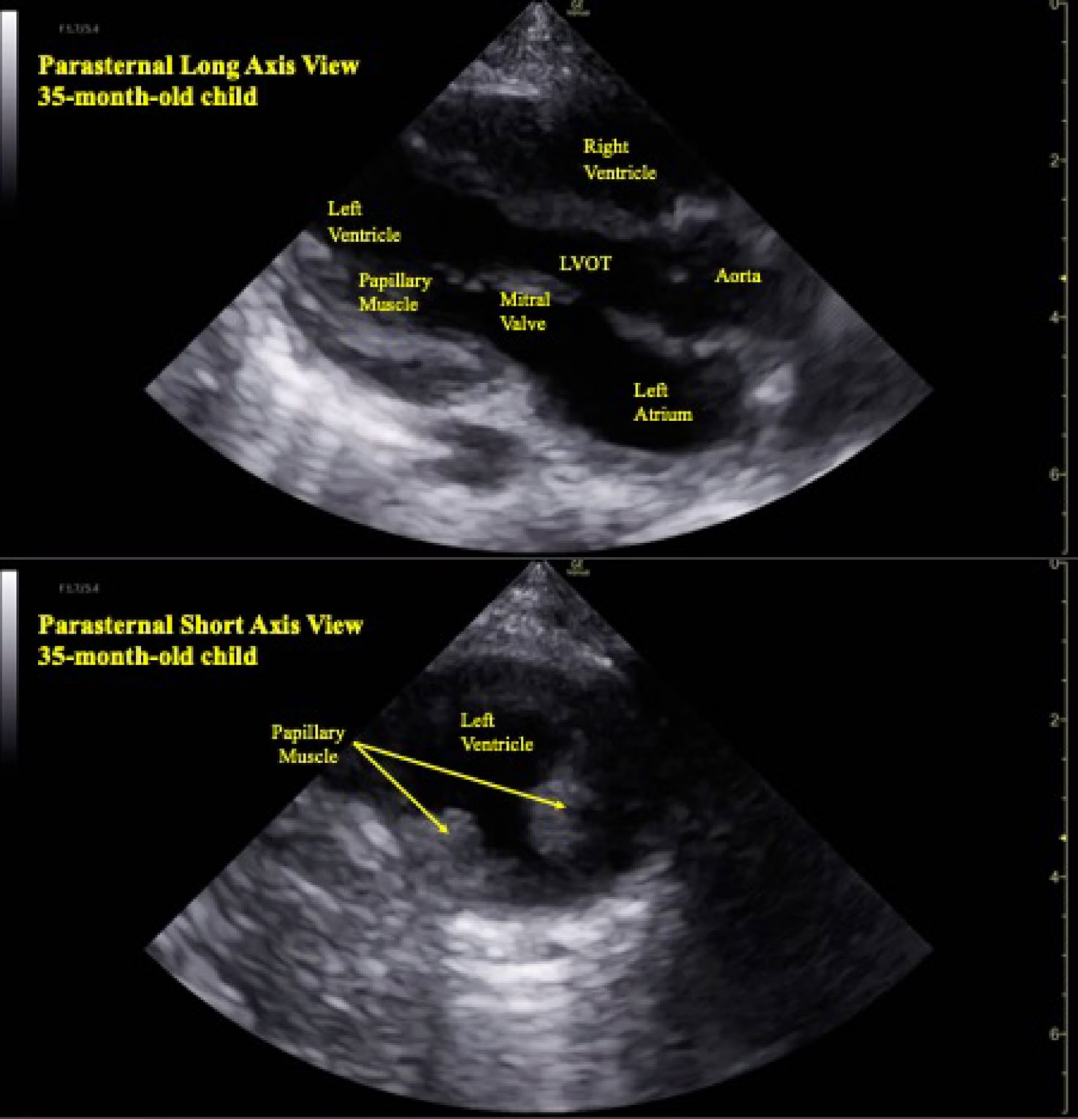
Representative parasternal long-axis and short-axis echocardiographic images for identifying the position of the left ventricle. Parasternal long-axis view and short-axis transthoracic echocardiographic images clearly visualize the left ventricle, left ventricular outflow tract, and papillary muscles, aiding in accurate identification of left ventricular position. LVOT, left ventricular outflow tract.

Transthoracic echocardiography was performed in the supine position using both left parasternal long-axis and short-axis views. To ensure accurate identification of the left ventricular position and confirmation of the intercostal space level, both views were obtained for each measurement. For cooperative participants, measurements were obtained during maximal inspiration and maximal expiration as instructed. For uncooperative participants, respiratory phases were visually assessed, and measurements were taken by estimating the points of maximal inspiration and expiration. The probe was positioned at the intercostal space where the image of the left ventricle including both papillary muscles and the left ventricular outflow tract was most clearly visualized during both maximal inspiration and expiration.(Fig. 2) Each subject underwent 5 measurements at inspiration and 5 at expiration, and the position of the left ventricle was recorded accordingly. The imaging location was described as the number of intercostal spaces relative to the intermammary line.

All echocardiographic examinations were performed by pediatric emergency physicians who had received formal training in pediatric point-of-care ultrasound, and all images were reviewed and confirmed by a board-certified specialist with over 10 years of experience in ultrasound education.

The ultrasound systems used for echocardiography were GE Healthcare’s Venue Go™ equipped with 3Sc-RS phased array probes (1–5 MHz).

### Outcomes

The primary outcome of this study was the anatomical location of the left ventricle during expiration, as visualized by transthoracic echocardiography. The secondary outcome was the difference in the anatomical position of the heart between inspiration and expiration.

### Statistical analysis

Continuous variables such as age and weight were summarized using median and range. To evaluate the relationship between respiratory phase and the vertical position of the heart, a linear mixed-effects model was fitted, treating heart position as a continuous outcome. Time, sex, age, and weight were included as covariates. A random intercept for subject ID was included to account for repeated measurements within individuals. A p-value of < 0.05 was considered statistically significant. All statistical analyses were conducted using STATA/SE 18.0 software (StataCorp LP, College Station, TX, USA).

## Results

Eighteen pediatric patients were enrolled in the study. For each participant, five repeated measurements were obtained during both inspiration and expiration, resulting in a total of 180 measurements. The median age was 56.5 months (interquartile range, 34.3–63.5), and the median body weight was 16.1 kg (interquartile range, 13.7–21.0). Eight participants (44.4%) were male. Abdominal pain and fever were the most common presenting complaints (Table 1).

**Table 1:** Baseline characteristics of the study population (*n* = 18).

Variables	Value
Sex, male, n (%)	8 (44.4)
Age, months, median (IQR)	56.5 (34.3–63.5)
Weight, kg, median (IQR)	16.1 (13.7–21.0)
Chief complaints, n (%)	
Abdominal pain	6 (33.3)
Fever	5 (27.8)
Vomiting	2 (11.1)
Cytopenia	1 (5.6)
Chest pain	1 (5.6)
Anaphylaxis	1 (5.6)
Dyspnea	1 (5.6)
Visual disturbance	1 (5.6)

IQR, interquartile range.

Intra-subject variation was observed in 9 out of 18 participants (50.0%), typically involving a one intercostal space difference between within the same respiratory phase. All individual-level measurement data are provided in Supplementary Table S1.

**Fig. 3:**
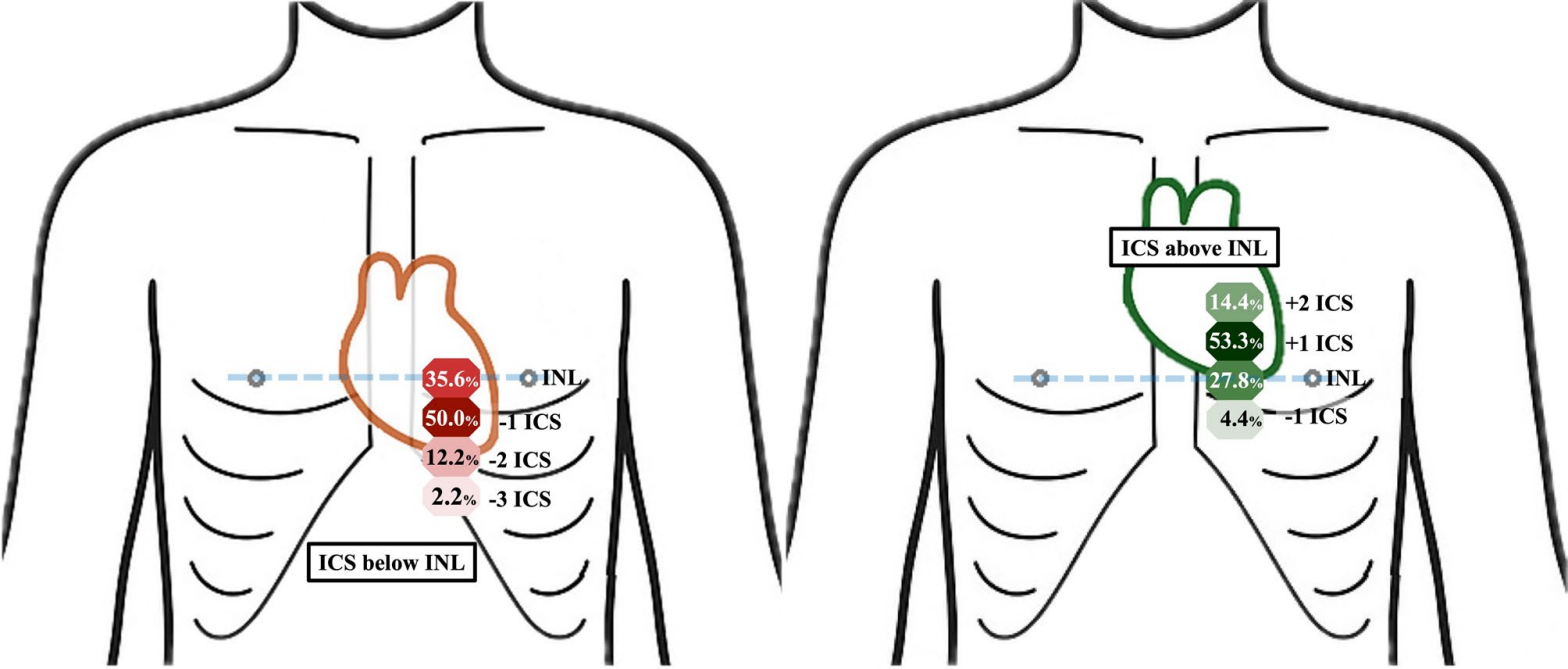
Schematic representation of left ventricular positional change during inspiration and expiration (90 measurements per phase). **(A)** Inspiration; **(B)** Expiration. A schematic chest diagram illustrates the positional change of the left ventricle across respiratory phases. The left ventricle descends below the inter-nipple line during inspiration and ascends to the level of or above the inter-nipple line during expiration. The blue line indicates the inter-nipple line. +1 and + 2 ICS denote the first and second intercostal spaces above the inter-nipple line; − 1, − 2, and − 3 ICS denote the first, second, and third intercostal spaces below. INL, inter-nipple line; ICS, intercostal space.

During inspiration, the left ventricle was predominantly located below the intermammary line, most often at the first intercostal space. In contrast, during expiration, it shifted upward and was most frequently observed above the intermammary line. The ventricle was never observed in upper intercostal spaces during inspiration, nor in lower intercostal spaces during expiration (Table 2; Fig. 3).

**Table 2:** Echocardiographic assessment of left ventricular position relative to the inter-nipple line during inspiration and expiration in pediatric patients (*n* = 180 measurements).

Respiration phase	Position of the Left Ventricle							Total
	3 ICS above	2 ICS above	1 ICS above	At INL	1 ICS below	2 ICS below	3 ICS below	
Inspiration, n (%)	0 (0.0)	0 (0.0)	0 (0.0)	32 (35.6)	45 (50.0)	11 (12.2)	2 (2.2)	90 (100.0)
Expiration, n (%)	0 (0.0)	13 (14.4)	48 (53.3)	25 (27.8)	4 (4.4)	0 (0.0)	0 (0.0)	90 (100.0)
Total, n (%)	0 (0.0)	13 (7.2)	48 (26.7)	57 (31.7)	49 (27.2)	11 (6.1)	2 (1.1)	180 (100.0)

ICS, intercostal space; INL, inter-nipple line.

The linear mixed-effects regression revealed that the heart was significantly lower during inspiration compared to expiration (β = −1.46, 95% CI: −1.91 to −1.00, *p* < 0.001). Time had no significant effect on heart position (β = 0.06, *p* = 0.262), and the phase-by-time interaction was also not significant (β = −0.04, *p* = 0.526), indicating that the positional difference between phases remained consistent across the respiratory cycle. Other covariates including sex (β = 0.15, *p* = 0.477), age (β = 0.00, *p* = 0.997), and body weight (β = −0.01, *p* = 0.761) were not significantly associated with the heart position.

## Discussion

The current study provides novel insight into the dynamic anatomical position of the left ventricle throughout the respiratory cycle in pediatric patients. Our findings demonstrated a significant cephalad displacement of the left ventricle during expiration, with frequent localization at or above the intermammary line. These observations challenge the conventional assumption that the heart consistently resides in the lower half of the sternum during resuscitation.

Previous studies, primarily based on static radiographic data, have suggested that the optimal chest compression site in pediatric patients lies in the lower rather than the mid portion of the sternum^9–12^. However, these investigations did not account for the dynamic respiratory mechanics that influence cardiac position or real-time anatomical changes that occur during cardiac arrest. Recent studies in adults have demonstrated significant discrepancies between guideline-recommended compression sites and actual cardiac anatomy, with investigations showing that compression location significantly influences both hemodynamic outcomes and the risk of thoracoabdominal injuries^15–17^. Our pediatric findings parallel these adult observations, suggesting that current compression landmarks may not optimally target the left ventricle across all clinical scenarios.

In contrast, by utilizing transthoracic echocardiography to assess left ventricular positioning throughout the respiratory cycle, our study provides a physiologically relevant perspective. Particularly, identification of the heart’s location at both inspiration and expiration offers critical information for optimizing chest compression landmarks during pediatric resuscitation, representing a significant advancement over previous static anatomical assessments.

Although intra-subject variation was observed in 9 of 18 participants (50.0%), typically manifesting as a one intercostal space difference within the same respiratory phase, this likely reflects physiological variability such as inconsistent respiratory effort, minor postural shift, or inherent cardiac motion. To account for this, each of the 180 measurements was treated as a distinct anatomical snapshot. The cephalad shift of the left ventricle during expiration remained consistent across all participants and time points, reinforcing the validity of our primary findings. Respiratory phase emerged as a significant determinant of the heart’s vertical position, with the heart consistently positioned lower during inspiration and relatively higher during expiration. This phase-dependent shift was independent of age, sex, and body weight, and the absence of a significant phase-by-time interaction suggests that it reflects a stable mechanical response rather than a transient fluctuation within the respiratory cycle.

### Respiration phase timing during CPR

In pediatric basic life support (BLS) without an advanced airway, a 15:2 compression-to-ventilation ratio is recommended^7^. Assuming a compression rate of 100 per minute (0.6 s per compression) and a ventilation duration of 2 s per breath, each cycle of 15 compressions and 2 ventilations takes approximately 13 s (9 s for compressions and 4 s for ventilations), resulting in non-ventilatory time comprising about 69% of the cycle. In contrast, during pediatric advanced life support with an advanced airway secured, continuous chest compressions are delivered with asynchronous ventilations at a rate of 20 breaths per minute (one every 3 s, each lasting 2 s), reducing non-ventilatory time to approximately 33% of the total cycle. This predominance of non-ventilatory time in pediatric BLS supports our focus on the cardiac position at end-expiration, which more accurately reflects the mechanical conditions present during the majority of the resuscitation effort.

Furthermore, during actual cardiac arrest, the heart may be positioned even more cephalad than our end-expiratory measurements suggest. Progressive lung collapse below functional residual capacity, potential consolidation, and absence of diaphragmatic tone during CPR could result in additional cephalad displacement of cardiac structures^21,22^. This consideration further supports the potential inadequacy of traditional lower sternal compression sites and emphasizes the need for reassessment of pediatric CPR landmarks.

### Clinical implications for different CPR scenarios

Our findings have differential implications based on CPR scenario. For compression-only CPR, which comprises approximately 50% of bystander-initiated pediatric out-of-hospital cardiac arrest cases^23^, the end-expiratory position data directly inform optimal hand placement. In these scenarios, positioning compressions at or above the intermammary line may more effectively target the left ventricle. For CPR with positive-pressure ventilation, the cardiac position likely varies between our measured inspiratory and expiratory positions, depending on ventilation parameters. The average position across respiratory phases in our study aligns more closely with current guidelines, supporting their continued use during ventilated CPR setting.

The selection of compression site requires careful balance. While our data suggest a more cephalad position during expiration, excessive cephalad placement risks LVOT compression and reduced cardiac output. While our data demonstrate a more cephalad position of the left ventricle during expiration, with 67.8% located at the first or second intercostal space above the intermammary line, the exact extent of this displacement was not quantified in millimeters. Our findings suggest that compressions at or slightly above the intermammary line may be more likely to target the left ventricular chamber during the expiratory phase, though further studies with direct compression assessment are needed to confirm optimal hand placement. Additionally, a more cephalad compression site could theoretically reduce the risk of subdiaphragmatic organ injury, though this potential benefit requires further investigation.

Accurate palpation of the lower half of the sternum during CPR is inherently challenging, especially in infants and young children^24^. As an alternative, the intermammary line has been proposed as a reliable external landmark to guide hand placement^25^. This approach minimizes the risk of inadvertent compression over the xiphoid process or upper abdominal organs, thereby reducing the potential for iatrogenic injuries. Notably, in prepubertal pediatric patients, the nipples are typically aligned with the fourth to fifth intercostal spaces, further validating the intermammary line as an appropriate anatomical reference. Accordingly, the present study evaluated the position of the left ventricle relative to the intermammary line rather than relying exclusively on sternum-based landmarks.

### Special considerations for the hyperinflation state

Special consideration is warranted for conditions causing hyperinflation, such as severe asthma leading to hypoxic cardiac arrest. In these cases, the heart may remain in a more caudal position due to persistent lung hyperexpansion, potentially making traditional compression sites more appropriate. This highlights the need for condition-specific CPR strategies.

However, caution must be exercised in generalizing these findings to all resuscitation contexts. In clinical practice, ventilation is often provided during CPR, and hyperventilation, which is characterized by rapid or deep breaths, is frequently observed^26^. Such ventilation practices can maintain the lungs in a partially inflated state, altering thoracic geometry and potentially shifting the heart’s position caudally compared to the end-expiratory state.

This study has some limitations. First, the sample size was relatively small (*n* = 18), primarily due to strict inclusion criteria, the impact of the COVID-19 pandemic, and the challenges of obtaining informed consent in a pediatric emergency setting. However, despite the limited number of participants, the results demonstrated high consistency, suggesting that the findings are robust and may be generalizable within the studied population. Second, since young infants are unable to follow instructions, the sonographer was required to actively track and scan the heart during respiration, which introduces potential variability depending on both the operator and the subject. Nevertheless, in this study, all ultrasound examinations were performed by a single experienced pediatric emergency physician, which likely contributed to the consistency and reliability of the measurements. Third, respiratory variability led to inconsistencies in left ventricular positioning between inspiration and expiration in some irritable or uncooperative participants, even within the same subject. To mitigate this issue, multiple measurements were obtained, thereby reducing the impact of outliers and enhancing the validity of the data.

## Conclusion

In this study, we identified a significant cephalad shift in the anatomical position of the left ventricle at end-expiration in pediatric patients using transthoracic echocardiography. While our end-expiratory findings are most relevant to compression-only CPR, meaning no respiratory support, the comprehensive respiratory cycle data we provide contributes to understanding optimal compression strategies across various CPR scenarios. These findings suggest that respiratory dynamics should be considered when determining the optimal chest compression site, particularly in infants and young children. To further validate these findings, large-scale studies targeting pediatric participants capable of controlled respiration are needed.

## Supplementary Information

Below is the link to the electronic supplementary material.

Supplementary Material 1

## Data Availability

The datasets generated and analyzed during the current study are included in the supplementary material.

## References

[1] Girotra, S. et al. Survival trends in pediatric in-hospital cardiac arrests: an analysis from get with the Guidelines-Resuscitation. *Circ. Cardiovasc. Qual. Outcomes*. **6**, 42–49 (2013). 23250980 10.1161/CIRCOUTCOMES.112.967968 PMC3555689

[2] Holmberg, M. J. et al. Trends in survival after pediatric In-Hospital cardiac arrest in the united States. *Circulation* **140**, 1398–1408 (2019). 31542952 10.1161/CIRCULATIONAHA.119.041667 PMC6803102

[3] Berg, R. A. et al. Incidence and outcomes of cardiopulmonary resuscitation in PICUs. *Crit. Care Med.* **44**, 798–808 (2016). 26646466 10.1097/CCM.0000000000001484 PMC4809365

[4] Fink, E. L. et al. Unchanged pediatric out-of-hospital cardiac arrest incidence and survival rates with regional variation in North America. *Resuscitation* **107**, 121–128 (2016). 27565862 10.1016/j.resuscitation.2016.07.244 PMC5037038

[5] Kitamura, T. et al. Nationwide improvements in survival from out-of-hospital cardiac arrest in Japan. *Circulation* **126**, 2834–2843 (2012). 23035209 10.1161/CIRCULATIONAHA.112.109496

[6] Straney, L. D. et al. Trends in PICU admission and survival rates in children in Australia and new Zealand following cardiac arrest. *Pediatr. Crit. Care Med.* **16**, 613–620 (2015). 25901547 10.1097/PCC.0000000000000425

[7] Topjian, A. A. et al. Part 4: pediatric basic and advanced life support: 2020 American heart association guidelines for cardiopulmonary resuscitation and emergency cardiovascular care. *Circulation* **142** (16 Suppl 2), S469–S523 (2020). 33081526 10.1161/CIR.0000000000000901

[8] Hwang, S. O. et al. Compression of the left ventricular outflow tract during cardiopulmonary resuscitation. *Acad. Emerg. Med.* **16**, 928–933 (2009). 19732038 10.1111/j.1553-2712.2009.00497.x

[9] Finholt, D. A. et al. The heart is under the lower third of the sternum: implications for external cardiac massage. *Am. J. Dis. Child.* **140**, 646–649 (1986). 3717100 10.1001/archpedi.1986.02140210044022

[10] Orlowski, J. P. Optimum position for external cardiac compression in infants and young children. *Ann. Emerg. Med.* **15**, 667–673 (1986). 3706857 10.1016/s0196-0644(86)80423-1

[11] Phillips, G. W. & Zideman, D. A. Relation of infant heart to sternum: its significance in cardiopulmonary resuscitation. *Lancet* **1**, 1024–1025 (1986). 2871296 10.1016/s0140-6736(86)91284-5

[12] Clements, F. & McGowan, J. Finger position for chest compressions in cardiac arrest in infants. *Resuscitation* **44**, 43–46 (2000). 10699699 10.1016/s0300-9572(99)00165-3

[13] Chu, S., Sun, J., Cheng, C., Ma, M. & Chiang, W. Abstract Or115: execution of transesophageal echocardiography in cardiopulmonary resuscitation for patients with out-of-hospital cardiac arrest (EXECT-CPR): a clustered randomized clinical trial. *Circulation* **150** (Suppl_1), AOr115 (2024).

[14] Park, G. Y., Oh, W. S., Chon, S. B. & Kim, S. The maximum diameter of the left ventricle May not be the optimum target for chest compression during cardiopulmonary resuscitation: a preliminary, observational study challenging the traditional assumption. *J. Cardiothorac. Vasc Anesth.* **34**, 383–391 (2020). 31585685 10.1053/j.jvca.2019.07.005

[15] Rolston, D. M. et al. Left of sternum compressions are associated with higher systolic blood pressure than lower half of sternum compressions in cardiac arrest. *Resuscitation* **206**, 110466 (2025). 39672254 10.1016/j.resuscitation.2024.110466

[16] Kim, H. et al. Optimum chest compression point might be located rightwards to the maximum diameter of the right ventricle: a preliminary, retrospective observational study. *Acta Anaesthesiol. Scand.* **64**, 1002–1013 (2020). 32196631 10.1111/aas.13577

[17] Gould, J. et al. Comparing sternal versus left-sided chest compressions for thoracoabdominal injuries and compression biomechanics: a clinical-grade cadaver study. *Resusc. Plus*. **21**, 100865 (2025). 39897062 10.1016/j.resplu.2025.100865 PMC11786900

[18] Park, M., Oh, W. S., Chon, S. B. & Cho, S. Optimum chest compression point for cardiopulmonary resuscitation in children revisited using a 3D coordinate system imposed on CT: a retrospective, cross-sectional study. *Pediatr. Crit. Care Med.* **19**, e576–e584 (2018). 30395117 10.1097/PCC.0000000000001679

[19] You, Y. Optimum location for chest compressions during two-rescuer infant cardiopulmonary resuscitation. *Resuscitation* **80**, 1378–1381 (2009). 19811866 10.1016/j.resuscitation.2009.08.013

[20] Cordioli, R. L. et al. Impact of ventilation strategies during chest compression. An experimental study with clinical observations. *J. Appl. Physiol.* **120**, 196–203 (2016). 26586906 10.1152/japplphysiol.00632.2015

[21] Shechter, G., Ozturk, C., Resar, J. R. & McVeigh, E. R. Respiratory motion of the heart from free breathing coronary angiograms. *IEEE Trans. Med. Imaging*. **23**, 1046–1056 (2004). 15338737 10.1109/TMI.2004.828676 PMC2494710

[22] Jagsi, R. et al. Respiratory motion of the heart and positional reproducibility under active breathing control. *Int. J. Radiat. Oncol. Biol. Phys.* **68**, 253–258 (2007). 17448878 10.1016/j.ijrobp.2006.12.058 PMC1865529

[23] Naim, M. Y. et al. Compression-only versus rescue-breathing cardiopulmonary resuscitation after pediatric out-of-hospital cardiac arrest. *J. Am. Coll. Cardiol.* **78**, 1042–1052 (2021). 34474737 10.1016/j.jacc.2021.06.042

[24] Yeung, J. et al. Basic life support providers’ assessment of centre of the chest and inter-nipple line for hand position and their underlying anatomical structures. *Resuscitation* **82**, 190–194 (2011). 21075499 10.1016/j.resuscitation.2010.10.008

[25] Birkenes, T. S., Myklebust, H. & Kramer-Johansen, J. New pre-arrival instructions can avoid abdominal hand placement for chest compressions. *Scand. J. Trauma. Resusc. Emerg. Med.* **21**, 47 (2013). 23799963 10.1186/1757-7241-21-47 PMC3694465

[26] Chapman, J. D., Geneslaw, A. S. & Babineau, J. Sen. A. I. Improving ventilation rates during pediatric cardiopulmonary resuscitation. *Pediatrics* **150**, e2021053030 (2022). 36000325 10.1542/peds.2021-053030

