# Supplementary material for: Reassessing chest compression sites in pediatric cardiopulmonary resuscitation without ventilatory support using echocardiographic assessment: a prospective observational study
Source: Sci Rep. 2025 Nov 6;15:38984. doi: 10.1038/s41598-025-22823-4 (PMC12592381; doi:10.1038/s41598-025-22823-4)
Supplement: Supplementary file 1 — Supplementary Material 1 [file 41598_2025_22823_MOESM1_ESM.pdf]

Reassessing Chest Compression Sites in Pediatric Cardiopulmonary Resuscitation Without Ventilatory Support Using Echocardiographic Assessment: A Prospective Observational Study

Dongbum Suh[1,2], Jin Hee Lee[1,2,*], Hyuksool Kwon[1], Mi Jin Kim[3]

[1] Department of Emergency Medicine, Seoul National University Bundang Hospital, Seoul National University College of Medicine, Gyeonggi-do, Republic of Korea

[2] Research Center for Disaster Medicine, Seoul National University College of Medicine, Seoul, Republic of Korea

[3] Department of Emergency Medicine, Seoul National University Hospital, Seoul, Republic of Korea

[*] Corresponding author: Jin Hee Lee, MD. PhD

E-mail: gienee@snubh.org

Table S1. Individual case measurements of left ventricular position during inspiration and expiration

Case ID	Sex	Age (month)	Weight (kg)	Inspiration					Expiration				
				1	2	3	4	5	1	2	3	4	5
1	M	33	16.0	-1*	-1	-1	-1	-1	1	1	1	1	1
2	F	60	14.4	-2	-2	-2	-1	-2	1	1	1	1	1
3	F	70	22.3	-2	-2	-2	-3	-3	1	1	1	1	1
4	F	58	16.0	-1	-1	-1	-1	-1	2	2	2	2	2
5	M	35	12.0	-1	-1	-1	-1	-1	1	1	1	1	1
6	F	64	19.0	-1	-1	-2	-2	-1	1	1	1	1	1
7	F	35	12.0	-1	-1	-1	-1	-1	1	1	2	1	1
8	M	26	13.5	-1	-1	0	0	0	1	1	1	2	1
9	M	62	21.0	-1	-2	-1	-1	-2	2	2	2	2	2
10	F	61	17.4	0	0	0	0	0	0	1	1	0	1
11	M	55	22.0	-1	0	-1	-1	0	0	0	0	1	0
12	M	72	24.0	-1	-1	0	-1	0	1	1	1	1	1
13	F	34	16.0	0	0	0	0	0	1	1	2	1	1
14	M	75	24.0	0	0	0	0	0	0	0	0	0	0
15	M	66	21.0	-1	-1	-1	-1	-1	-1	0	0	1	1
16	F	12	11.2	0	0	0	0	0	0	0	0	0	0
17	F	23	12.3	-1	-1	-1	-1	-1	-1	-1	0	-1	0
18	F	45	16.1	0	0	0	0	0	0	0	0	0	0

*The numeric values indicate the relative position of the left ventricle to the inter-nipple line on ultrasound. A value of 0 indicates that the left ventricle was visualized at the inter-nipple line; +1, +2, and +3 indicate visualization one, two, and three intercostal spaces above the inter-nipple line, respectively; −1, −2, and −3 indicate visualization one, two, and three intercostal spaces below the inter-nipple line, respectively.
